# Effects of different wavelengths of invasive laser acupuncture on chronic non-specific low back pain: a study protocol for a pilot randomized controlled trial

**DOI:** 10.1186/s13063-021-05038-6

**Published:** 2021-02-05

**Authors:** Jae-Hong Kim, Chang-Su Na, Gwang-Cheon Park, Jeong-Soon Lee

**Affiliations:** 1 Department of Acupuncture and Moxibustion Medicine, College of Korean Medicine, DongShin University, 185 Gunjae-ro, Naju City, 58245 Republic of Korea; 2 Clinical Research Center, DongShin University Gwangju Korean Medicine Hospital, 141 Wolsan-ro, Nam-gu, Gwangju City, 61619 Republic of Korea; 3 Department of Acupoint and Meridian, College of Korean Medicine, DongShin University, 185 Gunjae-ro, Naju City, 58245 Republic of Korea; 4 Department of Nursing, Christian College of Nursing, 6 Baekseo-ro 70 beon-gil, Nam-gu, Gwangju City, 61662 Republic of Korea

**Keywords:** Chronic non-specific low back pain, Laser acupuncture, Randomized controlled trial, Study protocol

## Abstract

**Background:**

Chronic non-specific low back pain (CLBP) is a common musculoskeletal disorder for which patients seek complementary and alternative medical treatments, including laser acupuncture (LA). Invasive LA (ILA) involves the simultaneous application of invasive acupuncture treatment at acupoints and focused laser irradiation. The efficacy of ILA for CLBP remains controversial owing to the insufficient clinical trial data. We intend to obtain basic data regarding the efficacy and safety of ILA for CLBP by comparing the effects of different wavelengths of ILA on CLBP.

**Methods:**

This will be a prospective, patient-blinded, parallel-arm, single-center (DongShin University Gwangju Korean Medicine Hospital, Republic of Korea), pilot randomized controlled clinical trial. Forty-five participants with CLBP will be randomized in equal numbers into the control, 650-nm ILA (650 ILA), or 830-nm ILA (830 ILA) group. The control group will receive sham ILA for 10 min and real electroacupuncture (EA) for 10 min. The 650 and 830 ILA groups will receive real ILA (i.e., 650 ILA group, 650-nm wavelength; 830 ILA group, 830-nm wavelength) for 10 min and real EA for 10 min once/day, twice a week for 4 weeks, at bilateral Shenshu (BL23), Qihaishu (BL24), Dachangshu (BL25), and Huantiao (GB30). The primary outcome will be an improvement in pain intensity assessed using the visual analog scale. Scores in the Korean version of the Oswestry Disability Index and the European Quality of Life Five Dimension Five Level scale will be recorded as secondary outcome measures. All scores will be recorded at baseline (before intervention), 4 weeks after the first intervention (at the end of the intervention), and 4 weeks after completion of the intervention.

**Discussion:**

The study is expected to provide preliminary evidence regarding the efficacy, safety, and usefulness of ILA for the treatment of CLBP.

**Trial registration:**

This trial was registered with the Clinical Research Information Service (registration No. KCT0004610; http://cris.nih.go.kr). Registered on 7 January 2020.

**Supplementary Information:**

The online version contains supplementary material available at 10.1186/s13063-021-05038-6.

## Background

Chronic non-specific low back pain (CLBP) is one of the most common, expensive, and disabling musculoskeletal conditions. CLBP is defined as pain and soreness, muscle tension, or stiffness in the lumbosacral area of the spine that does not have a specific underlying pathological cause and persists for > 3 months [1–3]. Functional limitations and consequent disability cause a heavy economic burden and poor quality of life in individuals and the society. In the USA alone, the expenditure on low back pain (LBP) has been estimated to be at least $100 billion per year [4]. A previous study reported the burden of chronic back pain in the general population and underscored its correlation with quality of life [5].

A cohort study demonstrated that > 51% of patients with LBP received complementary and alternative medicine (CAM) therapy during their 1-year follow-up [6]. In CAM, acupuncture, herbal medicine, thermal therapy, and spinal manipulative therapy have been used in patients with CLBP [7, 8]. Among CAM therapies, acupuncture is one of the most popular options [9]. On the basis of 5 systematic reviews (1 of high quality, 2 of moderate quality, and 2 of low quality), acupuncture, used either in isolation or as an adjunct to conventional therapy, provides short-term improvements in pain and function in patients with chronic LBP [10].

Low-level laser therapy (LLLT) is a noninvasive light source treatment that emits no heat, sound, or vibrations, but may act on cells via non-thermal or photochemical reactions [11] and can promote wound healing, peripheral nerve regeneration, pain relief, and further reduction of inflammation [12]. Laser acupuncture (LA), which involves focused irradiation at specific points, that is, at the most common traditional acupuncture points, with a low-intensity laser, has been commonly used over the last 35 years [13, 14]. Although LA treatment is a subgroup of LLLT, it is considered to be a separate form of treatment. Instead of using the direct effect of light on tissues to initiate a physiological response, the selection of points is based on a diagnostic and therapeutic paradigm defined by acupuncture theories [13, 15]. Different wavelengths, energy doses, and acupoints affect the effects of laser acupuncture [16–18].

A meta-analysis of randomized controlled trials (RCTs) of LLLT (including LA) for CLBP reported a moderate quality of evidence (GRADE) to support a clinically important benefit of LLLT for CLBP in the short term, which was only seen after higher laser-dose interventions and in participants with a shorter duration of back pain [14]. However, a couple of RCTs reported that noninvasive LA did not show significant effects as compared with sham lasers in patients with LBP [13, 19].

Noninvasive LA is applied to the skin and can be used as an alternative to needles through the use of laser-emitting devices. Invasive LA (ILA) involves the simultaneous application of invasive acupuncture treatment at acupoints and focused laser irradiation using a laser machine connected to an acupuncture needle consisting of an optical fiber-coupled laser diode. Previous studies showed that ILA combined with other treatments has significant effects on neuropathic pain and rheumatoid arthritis in rat models [20, 21]. However, relatively little evidence was obtained from clinical trials regarding the use of ILA for CLBP, especially rigorous randomized controlled clinical trials reporting on the efficacy of ILA. Therefore, we intend to obtain basic data regarding the efficacy and safety of ILA for CLBP by comparing the effects of different wavelengths of ILA.

## Methods/design

### Aims

This study will investigate the efficacy of ILA in the treatment of CLBP by comparing the effects of different wavelengths of ILA (650 and 830 nm) in terms of improvements in pain intensity, functional status, and patient quality of life.

### Hypothesis

The null hypothesis is that different wavelengths of ILA will provide similar improvements in pain intensity, functional status, and quality of life in patients with CLBP.

### Study design and setting

This manuscript was written in accordance with the SPIRIT (Standard Protocol Items: Recommendations for Interventional Trials) and CONSORT (Consolidated Standards Of Reporting Trials) 2010 guidelines [22, 23] (Additional file 1).

This study was a prospective, patient-blinded, parallel-arm, single-center (DongShin University Gwangju Korean Medicine Hospital, Republic of Korea), pilot randomized controlled clinical trial with a 1:1:1 allocation ratio. A total of 45 participants who met the inclusion criteria will be randomly allocated to the control, 650-nm ILA (650 ILA), or 830-nm ILA (830 ILA) group (*n* = 15 in each group). Participants in the control group will receive sham ILA for 10 min and real electroacupuncture (EA) for 10 min, while those in the 650 and 830 ILA groups will receive real ILA (i.e., 650 ILA group, 650-nm wavelength; 830 ILA group, 830-nm wavelength) and real EA both for 10 min. Participants will receive treatment once/day, twice/week for 4 weeks, at bilateral Shenshu (BL23), Qihaishu (BL24), Dachangshu (BL25), and Huantiao (GB30).

The primary outcome will be improvements in pain intensity assessed using the visual analog scale (VAS). The secondary outcome measures will include changes in scores in the Korean version of the Oswestry Disability Index (ODI) and the European Quality of Life Five Dimension Five Level scale (EQ-5D-5L). All scale scores will be recorded at baseline (before intervention), 4 weeks after the first intervention (i.e., at the end of the intervention), and 4 weeks after completion of the intervention.

**Fig. 1:**
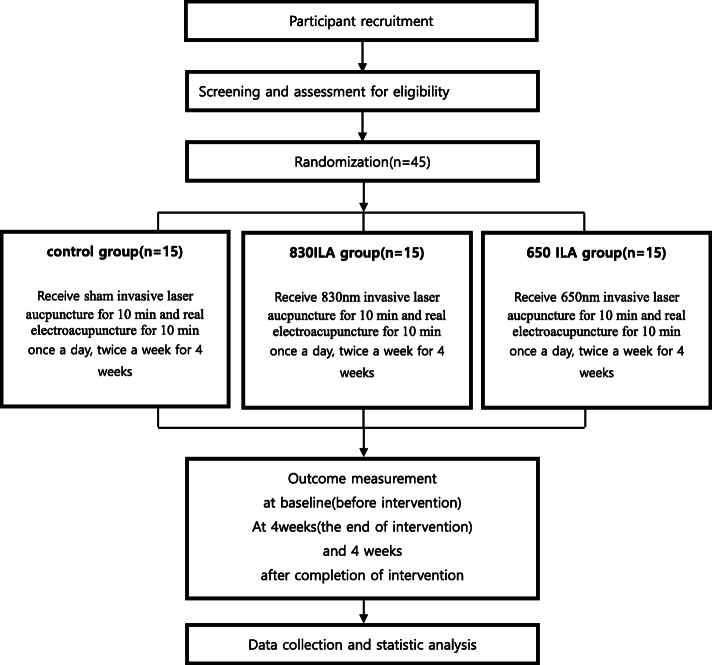
Study design flow chart

**Fig. 2:**
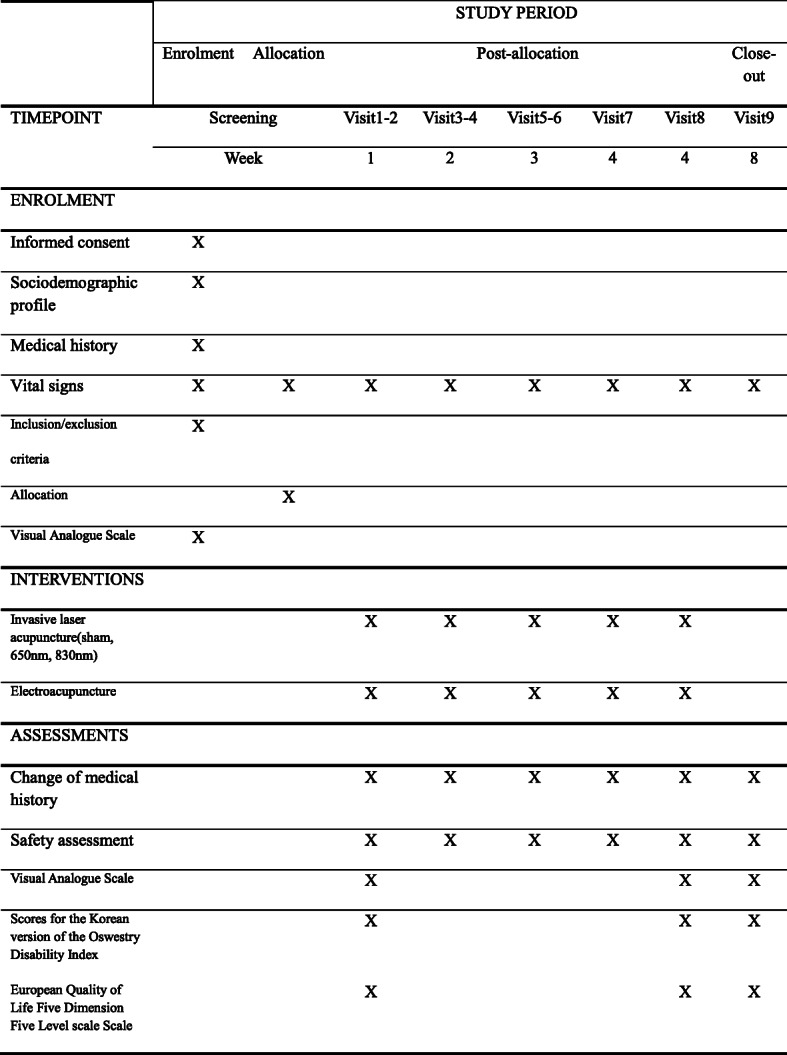
Standard Protocol Items: Recommendations for Interventional Trials Statement (SPIRIT). The figure shows the enrolment, interventions, and data collection

This study protocol complies with the principles of the Declaration of Helsinki and Korean Good Clinical Practice guidelines and was approved by the Ministry of Food and Drug Safety (Medical Device Clinical Trial Plan approval No. 1039). The trial was registered with the Clinical Research Information Service (registration No. KCT0004610; registration date: January 7, 2020; http://cris.nih.go.kr). The study design is summarized in Figs. 1 and 2.

### Participant recruitment

Participants will be recruited at the DongShin University Gwangju Korean Medicine Hospital, Republic of Korea. The study will be advertised through local newspapers, the Internet, and posters in communities and hospitals. Interested individuals will receive instructions for clinical trial participation through phone calls or visits to our hospital. When interested individuals visit the clinical research center at DongShin University Gwangju Korean Medicine Hospital, they will receive an explanation about the study from the clinical research coordinator (CRC) and will be asked to voluntarily sign an informed consent form prior to participation.

To facilitate participation in the study, the CRC will adjust the treatment and evaluation schedules of each individual participant. Every time the participants visit, the CRC will explain the schedule for the following visit and will remind the participant of the schedule by phone on the day before the appointment. The CRC will continuously monitor the medical condition of the enrolled participants to ensure their adherence to the intervention protocols.

### Inclusion criteria

Participants who meet all of the following criteria will be included in this trial: (1) aged between 19 and 70 years, (2) has CLBP lasting for at least the previous 3 months, (3) scored ≥ 40 points on a 100-mm VAS for pain at the time of screening, (4) has adequate level of Korean language proficiency for the reliable completion of all study assessments, and (5) voluntarily provides informed consent.

### Exclusion criteria

The exclusion criteria are as follows: (1) radicular pain or progressive neurological deficits; (2) diagnosis of a serious spinal pathology (cancer, recent vertebral fracture, spinal infection, or inflammatory spondylitis); (3) presence of a serious chronic disease (cancer, severe cardiovascular, cerebrovascular, liver, kidney disease, or diabetic neuropathy); (4) history of treatment for alcohol/drug dependency or mental illness (schizophrenia, dementia, or epilepsy) in the 6 months preceding enrollment; (5) LBP not caused by a spinal or soft tissue disease (trauma, ankylosing spondylitis, fibromyalgia, rheumatoid arthritis, or gout); (6) presence of contradictions for LA or EA, such as blood clotting abnormalities (hemophilia), severe skin disease in the lumbar region, presence of metallic devices in the lumbar spine, or presence of electronic medical devices (pacemaker); (7) previous lumbar spinal surgery within a year or scheduled procedures during the study; (8) pregnancy or planning to become pregnant; and (9) concurrent participation in other clinical trials.

### Dropout criteria

Participants will be dropped from the trial under the following conditions: (1) < 75% compliance with the protocol procedures (participating in < 6 of the 8 scheduled treatment sessions); (2) occurrence of a serious adverse event (SAE); (3) reluctance to continue the trial; (4) incomplete data that could influence the trial; (5) large errors in protocol or significant deviations in implementation; or (6) if the principal investigator (PI) or institutional review board (IRB) decides to terminate their participation in the trial.

### Ethical considerations

This study (protocol ver. 1.2) was approved by the IRB of DongShin University Gwangju Korean Medicine Hospital, Republic of Korea (approval NO: DSUOH-2019-004; date: April 17, 2020). The purpose and potential risks of this clinical trial will be fully explained to the participants and their families. All participants will be asked to provide written informed consent before participation.

### Randomization

After the acquisition of written informed consent, the practitioners who will be performing the intervention will conduct a screening interview. Then, the assessor will perform baseline measurements for participants who meet the inclusion criteria. The 45 enrolled participants will be immediately assigned serial numbers generated using the SPSS version 21 software (IBM Corp., Armonk, NY, USA) and randomly allocated to 1 of the 3 study groups (*n* = 15 per group). The serial number codes will be inserted in opaque envelopes that will be sealed and stored in a double-locked cabinet; these will be opened by the PI or practitioners who will perform the intervention in the presence of the patient and a guardian.

### Implementation

The CRC will generate the allocation sequence, enroll participants, and assign participants to the interventions.

### Blinding

Owing to the nature of LA treatment, a double-blind study design cannot be adopted. Therefore, we will adopt a patient-blinded trial procedure using sham LA. During the course of this clinical trial, the assessor will have no contact with the participants other than at the time of assessment. Furthermore, unblinding will not be permitted under any circumstances. However, if an SAE occurs, unblinding will be permitted after an agreement between all the researchers involved. To prevent selection, performance, and attrition biases due to the unblinded participants and practitioners, this study will only involve individuals without conflicts of interest or preconceived positions. All the practitioners who will perform the interventions will receive training in clinical trials before participation. A statistician not involved in the design or execution of the clinical trial will analyze the final data.

### Interventions

The treatment will be administered by Korean medical doctors with 6 years of formal university training in Korean medicine and a license to administer treatments. To ensure strict adherence to the study protocol, the practitioners who will perform the interventions will undergo training together and use the same techniques (Table 1).

**Table 1:** Revised Standards for Reporting Intervention in Clinical Trials of Acupuncture (STRICTA)

	Item criteria	Description
1. Acupuncture rationale	1a) Style of acupuncture	Korean medicine therapy
	1b) Reasoning for treatment provided—based on historical context, literature sources, and/or consensus methods, with references where appropriate	1) Discussion among four doctors that practice Korean medicine (consensus) 2) Textbook of acupuncture and moxibustion medicine 3) Relevant articles [13, 14, 19, 24, 25] Selection of treatment regions based on textbooks, related papers, and expert discussions
	1c) Extent to which treatment varied	Standardized treatment
2. Details of needling	2a) Number of needle insertions per subject per session (mean and range where relevant)	8
	2b) Names (or location if no standard name) of points used (unilateral/bilateral)	BilateralShenshu (BL23), Qihaishu (BL24), Dachangshu (BL25), and Huantiao (GB30)
	2c) Depth of insertion, based on a specified unit of measurement or a particular tissue level	The depth of insertion will be 9–30 mm, depending on the location of the needle [26]. After insertion, the needles will be left in position for 20 min.
	2d) Responses sought	No de qi or muscle twitching—only sensation due to needle insertion
	2e) Needle stimulation	650-nm invasive laser, 830-nm invasive laser, and electrical stimulation
	2f) Needle retention time	20 min per session
	2 g) Needle type	Sterile, stainless steel, disposable acupuncture needle (external diameter, 0.3 mm; inner diameter, 0.15 mm; length, 30 mm) consisting of an optical fiber-coupled laser diode (i.e., 650 nm, InGaAIP; 830 nm, GaAIAs) and low-level laser
3. Treatment regimen	3a) Number of treatment sessions	8
	3b) Frequency and duration of treatment sessions	Once/day, twice/week for 4 weeks, 20 min per session
4. Other treatment components	4a) Details of other interventions administered to the acupuncture group	Participants in the control group will receive sham ILA for 10 min and real electroacupuncture (EA) for 10 min, while those in the 650 and 830 ILA groups will receive real ILA (i.e., 650 ILA group, 650-nm wavelength; 830 ILA group, 830-nm wavelength) for 10 min and real EA for 10 min.
	4b) Setting and context of treatment—including instructions to practitioners and information and explanations given to patients	Practitioner-patient conversation about the context of the treatment, life habits, and daily life management
5. Practitioner background	5a) Description of the participating acupuncturists	Korean medicine doctor with the following qualifications: 6 years of formal university training in Korean medicine and a license
6. Control or comparator interventions	6a) Rationale for the control or comparator in the context of the research question, with sources that justify the choice	○ Glazov G, Yelland M, Emery J. Low-dose laser acupuncture for non-specific chronic low back pain: a double-blind randomized controlled trial. J. Acupunct Med. 2014;32:116–123. ○Glazov G, Yelland M, Emery J. Low-level laser therapy for chronic non-specific low back pain: a meta-analysis of randomized controlled trials. J. Acupunct Med. 2016;34:328–341. ○Hwang MS, Heo KH, Cho HW, Shin BC, Lee HY, Heo I, Kim NK, Choi BK, Son DW, Hwang EH. EA as a complement to usual care for patients with non-acute pain after back surgery: a study protocol for a pilot randomized controlled trial. BMJ Open. 2015;5:e007031.
	6b) Precise description of the control or comparator; details for items 1–3 above with the use of sham acupuncture or any other type of acupuncture-like control	Participants in the control group will receive sham ILA for 10 min and real EA for 10 min, while those in the 650 and 830 ILA groups will receive real ILA (i.e., 650 ILA group, 650-nm wavelength; 830 ILA group, 830-nm wavelength) for 10 min and real EA for 10 min. The laser parameters will be as follows: pulse mode, pulse-type wave; output power, 20 mW; frequency, 50 Hz; and irradiated area, 0.15 × 0.15 mm^2^. EA will be applied with a biphasic waveform current, which is a compressional wave that combines an interrupted wave and a continuous wave, in triangular form, at a frequency of 50 Hz [25]. Sham ILA will be applied without any laser power output.

**Fig. 3:**
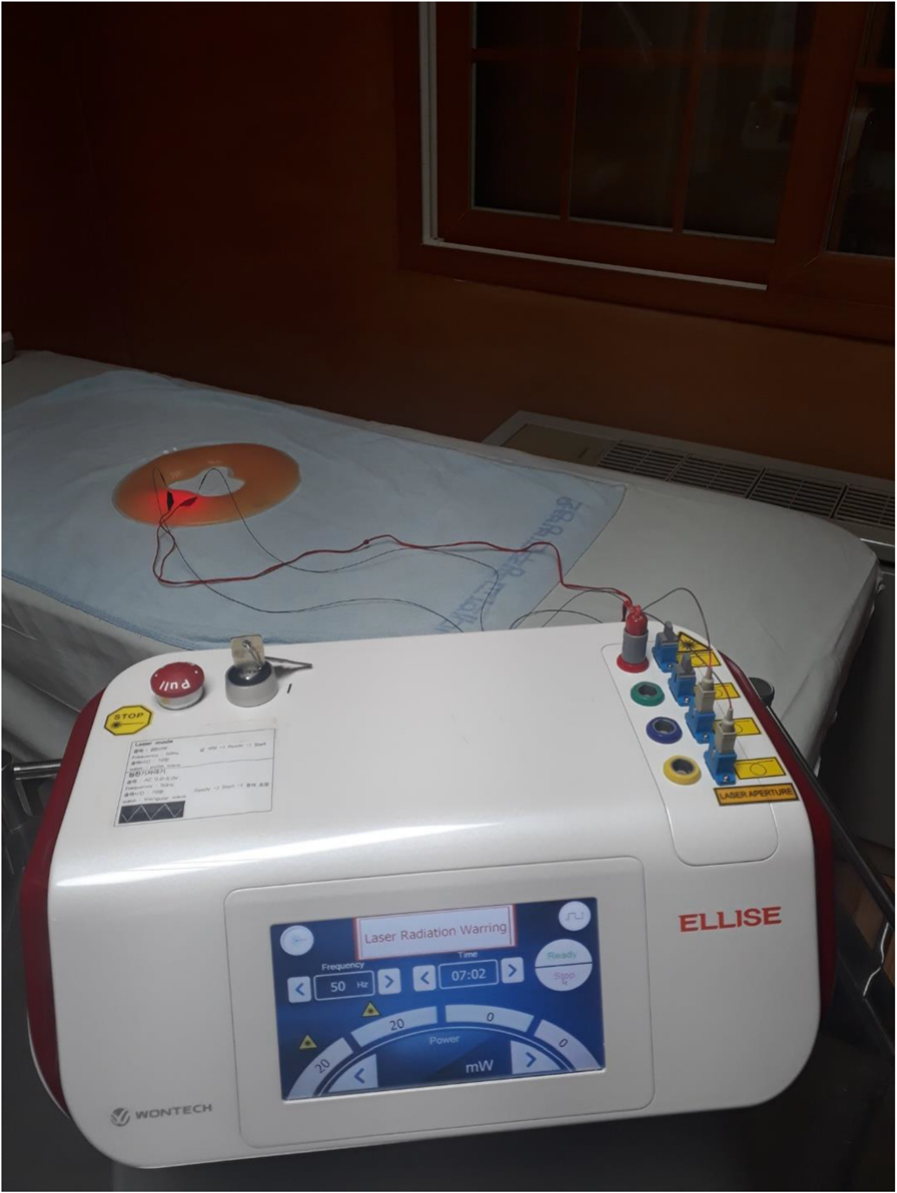
The intervention will be performed with medical device of invasive laser acupuncture needle, low-level laser, and electrical stimulator (Ellise)

ILA and EA will be performed with a medical device composed of a combination of a sterile, stainless steel, disposable acupuncture needle (external diameter, 0.3 mm; inner diameter, 0.15 mm; length, 30 mm), optical fiber-coupled laser diode (i.e., 650 nm, InGaAIP; 830 nm, GaAIAs), low-level laser, and electrical stimulator (Ellise; Wontech, Co., Ltd., Daejeon, Republic of Korea) which will be administered (Fig. 3). With participants in the prone position, the needles will be vertically inserted in Shenshu (BL23), Qihaishu (BL24), Dachangshu (BL25), and Huantiao (GB30) [24, 27]. The depth of insertion will be between 9 and 30 mm, depending on the location of the needle [26]. After insertion, the needles will be left in position for 20 min. Manual stimulation will not be used. Participants in the control group will receive sham ILA for 10 min and real EA for 10 min, while those in the 650 and 830 ILA groups will receive real ILA (650 ILA group, 650-nm wavelength; 830 ILA group, 830-nm wavelength) and real EA both for 10 min. Participants will receive treatment once/day, twice/week for 4 weeks. The laser parameters will be as follows: pulse mode, pulse-type wave; output power, 20 mW; frequency, 50 Hz; and irradiated area, 0.15 × 0.15 mm^2^. EA will be applied with a biphasic waveform current, which is a compressional wave that combines an interrupted wave and a continuous wave, in triangular form, at a frequency of 50 Hz [25]. On the basis of the previous RCTs to investigate the efficacy of LLLT on CLBP [13, 19], the control group will undergo the same procedures as the ILA group, but the laser will not be turned on. The acoustic functions of the equipment will be preserved to ensure blinding. No significant differences in observation, feeling, or sound should be found between the 3 groups during the procedure. Hence, all participants will be blinded to the group selection.

All participants should comply with the protocol procedures such as the allocated intervention and treatment schedules. However, the treatment schedule may be changed according to the judgment of the PI or the request of the participant.

During the clinical trial period, all participants will be allowed to continue the routine management regimens, existing medications (e.g., those for hypertension, diabetes, or hyperlipidemia), and medications for maintaining and improving their health status. However, they will not be permitted to engage in other treatments (pharmacological treatments, physical therapy, or CAM therapies) to ameliorate their CLBP symptoms. All medical devices will be inspected by the investigators, who will record the results of checkups in the management register.

### Outcome measurements

In accordance with the study objective, improvements in pain intensity assessed using the VAS will be considered the primary outcome, and the secondary outcomes will be the changes in ODI and EQ-5D-5L. VAS, ODI, and EQ-5D-5L scores will be recorded before intervention, at the end of intervention, and 4 weeks after the completion of the intervention. The primary end point will be the VAS score recorded at the end of the intervention.

The VAS is a 10-cm-long straight line marked at each end with the anchor labels “no pain” and “pain as bad as it could be” [28]. Participants will be asked to mark a point representing the severity of their pain. Scores are recorded in millimeters, and the total score ranges from 0 to 100 mm [29].

The ODI has become one of the principal condition-specific outcome measures used in the management of spinal disorders [30]. The validated Korean version of the ODI, which excludes sexual life items from the original ODI, contains 9 questions about daily activities, including inventories of pain intensity, personal care, lifting, walking, sitting, standing, sleeping, social life, and traveling. Each question is rated on a scale from 0 to 5 points, with higher scores indicating greater severe pain-related disability [31].

The validated Korean version of the EQ-5D includes generic questions about personal health-related quality of life and 5 dimensions pertaining to mobility, self-care, usual activities, pain/discomfort, and anxiety/depression. Each dimension has 3 response categories, namely “none,” “some,” and “extreme/unable to,” with lower scores indicating a better state of health [32, 33]. The EQ-5D-5L is a new version of the EQ-5D that includes 5 levels of severity (none, slight, moderate, severe, and extreme/unable) for each of the 5 EQ-5D dimensions [34].

### Incidence of AEs

AEs are undesirable and unintentional signs, symptoms, or diseases that appear during or after treatment in a clinical trial. Participants in this study will be required to voluntarily report any AEs. All AEs that occur during the trial will be documented. AEs that could occur in this study include skin irritation, bleeding, local hematoma, pallor, sweating or dizziness, fainting during acupuncture treatment, continuous severe pain for > 1 h after acupuncture treatment, objective worsening of existing symptoms, and undesirable and unintentional signs, symptoms, or diseases. The CRC will record all AEs in detail, including the time and date of occurrence, degree of severity, any measures related to the treatment of the AE, and any potential causal relationships between the treatment and the AE. All AEs will also be reported to the PI and relevant IRB. In case of SAEs, defined as AEs causing severe disability or malfunction, appropriate measures will be taken, and the incident will be immediately reported to the PI and relevant IRB. In cases in which an AE occurs because of the clinical trial, participants will notify the CRC and PI and will be compensated.

### Quality assurance

This protocol has been reviewed and revised several times by experts on acupuncture, orthopedics, statistics, and methodology. Before the trial, all the researchers will be requested to attend a series of training sessions, which will ensure that the personnel involved fully understand the trial protocol and standard operating procedures (SOPs) of the study. The data monitoring committee (DMC) will comprise the PI, CRC, and a researcher who will not be involved in the data collection aspect of this study. The DMC, which is independent from the sponsor and free from competing interests, will manage the data to ensure data validity. This study will be monitored by a clinical research associate (CRA), who will check all documents related to this study, including the case report forms (CRFs) and SOPs, and ensure that this clinical trial is conducted in accordance with the prescribed protocols and SOPs. Monitoring will be performed by an independent CRA, who will not be involved in any other aspect of the trial. In the event that the protocol described herein is revised, the revisions will require approval from the Ministry of Food and Drug Safety and the IRB of DongShin University Gwangju Korean Medicine Hospital.

### Sample size estimation

We had no preliminary study or adequate previous studies upon which to base sample size estimates. Therefore, we adopted a pilot study design. The appropriate sample size for two- or three-arm pilot studies is > 12 [35, 36]. Considering a dropout rate of 20%, we assigned 15 participants to each group (45 in total).

As our study is a pilot study, the sample size will not be sufficient for determining the efficacy of ILA for CLBP. Our study provides preliminary evidence for the efficacy and safety of ILA for CLBP.

### Statistical analysis

A statistician not involved in data collection will analyze the final data. We will perform a full analysis set (FA group) to assess the efficacy and a supplementary per-protocol analysis (PP group). We will compare the results of the statistical analyses between the FA and PP groups and confirm any statistically significant differences between the groups. If a significant difference exists between the PP and FA groups, the cause will be reviewed and reflected in the efficacy assessment. Missing values will be obtained using the “last observation carried forward” method. Interim analyses will not be performed. All statistical analyses will be performed using the SPSS version 21 software.

Baseline characteristics will be described and compared between the groups. Continuous data will be presented as means and standard deviations and compared using the independent *t* test or Mann-Whitney *U* test (nonparametric test). Categorical data will be presented as frequencies and percentages and compared using the chi-square or Fisher exact test.

Within each group, changes in VAS, ODI, and EQ-5D-5L scores at 4 weeks (the end of intervention) and 8 weeks (4 weeks after the end of intervention) after the start of intervention, relative to the baseline score, will be analyzed using a paired *t* test or Wilcoxon signed rank test (nonparametric test). Trends over time and time-by-treatment interactions will be explored using repeated-measure analysis of variance. The degrees of changes in the VAS, ODI, and EQ-5D-5L scores at each time point between the groups will be evaluated using the independent *t* test or Mann-Whitney *U* test (nonparametric test). Subanalyses will be performed according to participant age. All reported *p* values will be two-sided, with 95% confidence intervals. A *p* value < 0.05 will be considered statistically significant.

A safety assessment will be performed for all AEs that will occur during the study period. The incidence of AEs and SAEs will be summarized by group and analyzed using the chi-square or Fisher exact test.

### Confidentiality and data management

All identification records of the participants will be kept confidential. When the results of the study are published, the identification records will be accessible under IRB approval. All documents related to the trial, including CRFs, will be recorded and labeled with participant identification codes and will not reveal the name of the participants. The serial number codes will be stored in sealed, opaque envelopes and kept in a double-locked cabinet. All participant data will be recorded in Excel files by the CRC. All data will be checked by the CRC and rechecked by a researcher, both of whom will not be included in the data collection phase of the study to ensure the reliability of the data. Continuous data will be coded as data values, and categorical data will be coded as number sets for each item by a statistician not involved in the data collection phase of this clinical trial. The data entry and coding will be double-checked. Electronic data will be stored on a password-protected computer. Anyone who is not approved by the IRB will not be able to access the data. In addition, raw data (CRFs) will be stored in a cabinet until the end of the study. Written informed consent for the publication of the individual details and accompanying images will be obtained from the participants.

## Discussion

Although LA has long been used in the treatment of CLBP, the efficacy and optimal treatment method of LLLT (including LA) for CLBP remains controversial owing to insufficient evidence. We designed this study to investigate the efficacy of ILA and the effects of different wavelengths of ILA on CLBP. The design of this study, including the treatment method, schedules, and outcome measurements, is based on the design of several studies that evaluated various types of acupuncture treatment for LBP [10, 13, 14, 19, 24, 25, 27]. The two modalities of ILA (650 and 830 nm) are based on our previous studies that showed that ILA has significant effects on neuropathic pain [37] and osteoarthritis [38] in rat models and previous RCTs that investigated the efficacy of LLLT on CLBP [13, 14].

Changes in VAS pain score will be recorded as the primary outcome, while changes in ODI and EQ-5D-5L scores will be recorded as secondary outcomes. This study will evaluate the effects of different wavelengths of ILA on pain reduction, pain-related functional disability, and quality of life in patients with CLBP.

This protocol has some limitations. First, owing to the lack of adequate preliminary studies and limited funding, this survey was designed as a single-center pilot study with a small sample size. Second, while previous LA studies on CLBP used noninvasive LA [13, 14, 19], we will only use ILA. Third, the factors that affect the effects of LA are wavelength, energy dose, and selected acupoints; however, we will only investigate the effects of wavelength. Fourth, although participants will be blinded to the group allocation and all outcome measures will be measured and recorded by an independent researcher to minimize the risk of detection bias, the acupuncturists cannot be blinded to the group allocation owing to the nature of LA.

Nevertheless, the results of this study are expected to provide preliminary evidence regarding the usefulness, safety, and efficacy of ILA for the treatment of CLBP and the effects of different wavelengths of ILA, and thus will provide a foundation for further research.

### Dissemination policy

We will report the final data to the Ministry of Health and Welfare, Republic of Korea, through the Korea Health Industry Development Institute. We will also publish the results after the completion of the study.

### Trial status

Participants’ recruitment was terminated. We recruited participants between May 12, 2020, and September 17, 2020. The trial procedures are expected to be completed by the end of December 2020.

## Supplementary Information

**Additional file 1.** SPIRIT 2013 checklist: Recommended items to address in a clinical trial protocol and related documents*.

## Data Availability

The datasets used and/or analyzed during the present study are available from the corresponding author upon reasonable request.

## Abbreviations

AE: Adverse event
CAM: Complementary and alternative medicine
CLBP: Chronic non-specific low back pain
CONSORT: Consolidated Standards Of Reporting Trials
CRA: Clinical research associate
CRC: Clinical research coordinator
CRF: Case report form
DMC: Data monitoring committee
EA: Electroacupuncture
EQ-5D-5L: European Quality of Life Five Dimension Five Level scale
IRB: Institutional review board
ILA: Invasive laser acupuncture
LA: Laser acupuncture
LBP: Low back pain
LLLT: Low-level laser therapy
ODI: Oswestry Disability Index
PI: Principal investigator
RCTs: Randomized controlled trials
SAE: Serious adverse event
SOPs: Standard operating procedures
SPIRIT: Standard Protocol Items: Recommendations for Interventional Trials
VAS: Visual analog scale
